# Gross motor adaptation benefits from sleep after training

**DOI:** 10.1111/jsr.12961

**Published:** 2019-12-23

**Authors:** Kathrin Bothe, Franziska Hirschauer, Hans‐Peter Wiesinger, Janina M. Edfelder, Georg Gruber, Kerstin Hoedlmoser, Juergen Birklbauer

**Affiliations:** ^1^ Laboratory for Sleep, Cognition and Consciousness Research Centre for Cognitive Neuroscience University of Salzburg Salzburg Austria; ^2^ Department of Sport and Exercise Science University of Salzburg Salzburg Austria; ^3^ Department of Psychiatry and Psychotherapy Medical University of Vienna Vienna Austria

**Keywords:** gross motor learning, motor memory consolidation, rapid eye movement, sleep spindles

## Abstract

Sleep has been shown to facilitate the consolidation of newly acquired motor memories. However, the role of sleep in gross motor learning, especially in motor adaptation, is less clear. Thus, we investigated the effects of nocturnal sleep on the performance of a gross motor adaptation task, i.e. riding an inverse steering bicycle. Twenty‐six male participants (*M* = 24.19, *SD* = 3.70 years) were randomly assigned to a PM‐AM‐PM (*n* = 13) or an AM‐PM‐AM (*n* = 13) group, i.e. they trained in the evening/morning and were re‐tested the next morning/evening and the following evening/morning (PM‐AM‐PM/AM‐PM‐AM group) so that every participant spent one sleep as well as one wake interval between the three test sessions. Inverse cycling performance was assessed by speed (riding time) and accuracy (standard deviation of steering angle) measures. Behavioural results showed that in the PM‐AM‐PM group a night of sleep right after training stabilized performance (accuracy and speed) and was further improved over the subsequent wake interval. In the AM‐PM‐AM group, a significant performance deterioration after the initial wake interval was followed by the restoration of subjects' performance levels from right after training when a full night of sleep was granted. Regarding sleep, right hemispheric fast N2 sleep spindle activity was related to better stabilization of inverse cycling skills, thus possibly reflecting the ongoing process of updating the participants' mental model from “how to ride a bicycle” to “how to ride an *inverse* steering bicycle”. Our results demonstrate that sleep facilitates the consolidation of gross motor adaptation, thus adding further insights to the role of sleep for tasks with real‐life relevance.

## 1 INTRODUCTION

Motor learning is one of the most fundamental processes in everyday life. Furthermore, motor adaptation skills are needed for modifying and reoptimizing learned motor behaviour in response to environmental or internal changes. Recent research suggests that a brain network in the right hemisphere, including the right inferior parietal lobule, the anterior insula and the medial prefrontal cortex is particularly relevant for adaptation to these changes (Filipowicz, Anderson, & Danckert, 2016). Additionally, it has been shown that recently acquired motor memories are further facilitated “off‐line” during sleep. However, the vast majority of sleep studies in laboratory settings have been focused on fine motor adaptation learning (for review, see King, Hoedlmoser, Hirschauer, Dolfen, & Albouy, 2017). Although most activities in daily life (e.g. driving a car, riding a bike) require complex gross motor skills, data in this domain are still rare. Ecologically valid motor tasks are complex in nature, require a large number of skeletal muscles and are thus initially more difficult to master than fine motor tasks. Therefore, it has been stated that findings and model conceptions derived from fine motor learning cannot simply be transferred to gross motor skills (Wulf & Shea, 2002). So far, mainly our own studies investigated the role of sleep in gross motor adaptation learning. (a) In 2015, Hoedlmoser et al. reported that increases in N2 sleep spindle activity (SpA) and rapid eye movement (REM) duration during a post‐training diurnal nap decreased the ability of adults to ride an inverse steering bicycle after the nap. (b) For nocturnal sleep, Bothe et al. (2018) showed that, in adolescents, improved inverse steering accuracy was associated with an increase in N2 SpA and a decrease in REM duration from a control night to a test night, while improvements in speed were related to an increase in REM duration. Thus, a whole night of sleep as compared with a nap might facilitate gross motor adaptation instead of hindering it (Schönauer, Geisler, & Gais, 2014; Van Schalkwijk et al., 2017). Due to the underrepresentation of gross motor studies, particularly adaptation tasks, the main objective of the current study was to investigate whether, in adults, the acquisition and consolidation of a complex gross motor adaptation task, i.e. riding an inverse steering bicycle, is dependent on nocturnal sleep right after training (as compared with wakefulness). The inverse steering bicycle is a self‐built, conventional bike with a fixed gear ratio. The steering is constructed with two equal gear wheels so that the bicycle has to be controlled inversely by mirrored steering movements. According to Nishida and Walker (2007), daytime experience leads to local reactivations of recently acquired memory representations during sleep. N2 sleep spindles have repeatedly been linked to procedural memory consolidation (for review, see King et al., 2017). Taking into account the nature of the task, i.e. adaptation of a learned motor behaviour to external changes, we expected a learning‐related increase in N2 sleep SpA most likely to be seen across the right hemisphere. Because REM duration has been involved in previous gross motor adaptation studies (Bothe et al., 2018), we also expected an increase in REM duration to be associated with improved performance. Moreover, it has been suggested that phasic REM (i.e. REM containing characteristic rapid eye movements) and tonic REM (i.e. REM without characteristic rapid eye movements) episodes might serve different functions in the consolidation process: while tonic REM supports selective local replay and pattern separation of previously encoded information, phasic REM seems to be more important for the exchange of information between hippocampus and neocortex (Hutchison & Rathore, 2015). Lastly, spectral theta activity during REM has been linked to the reactivation and consolidation of previously encoded memories (Boyce, Glasgow, Williams, & Adamantidis, 2016; Fogel, Smith, & Cote, 2007; Schönauer et al., 2017). Thus, we additionally analysed spectral theta activity during tonic and phasic REM periods to see whether they are differently involved in the consolidation of our gross motor adaptation task.

## 2 MATERIALS AND METHODS

### 2.1 Subjects

**Figure 1:**
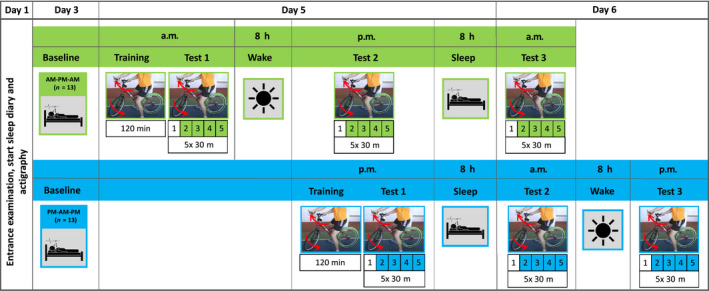
Study design. Participants were randomly assigned to an AM‐PM‐AM or a PM‐AM‐PM group: they either trained in the morning/evening and were re‐tested the next evening/morning and the following morning/evening, i.e. every participant spent a sleep as well as a wake retention interval between test sessions

Thirty‐five right‐handed, healthy men aged between 20 and 36 years (*M* = 24.77, *SD* = 4.03) were recruited and randomly assigned to either an AM‐PM‐AM (*n* = 16) or a PM‐AM‐PM (*n* = 19) group (Figure 1). Exclusion criteria included overweight (body mass index above 25), sleep and psychological disorders as well as medication or drug intake that could disturb sleep or cognitive abilities. Furthermore, professional/competitive cyclists and/or participants with pre‐experience in riding an inverse steering bicycle were excluded. To ensure that only subjects who had learned to handle the inverse steering bicycle were included in the analyses, we used five parameters to assess subjects' overall performance: (a) ability to ride three runs of 30 m without dismounting; (b) distance covered during the training session; as well as (c) steering accuracy, (d) riding time and (e) number of dismounts during TEST 1. Based on the factor scores of a factor analysis (Varimax orthogonal rotation; Table S1 Supporting Information), 26 subjects (AM‐PM‐AM: *n* = 13; PM‐AM‐PM: *n* = 13) were defined as learners (excluding the rest of the subjects from further analysis). This sample included all subjects being able to ride three runs of 30 m without dismounting and three further subjects who performed better than average at TEST 1. The classification was also confirmed by a hierarchical cluster analysis (based on Euclidean distance; Figure S1), separating learners and non‐learners into two distinct clusters. The study was performed in accordance with the Declaration of Helsinki and approved by the local ethics committee. Subjects gave their written informed consent before study inclusion and received 50 € for participation.

### 2.2 Experimental design

All participants underwent an entrance examination, including an evaluation of chronotype (Griefahn, Künemund, Bröde, & Mehnert, 2001), sleep quality (Buysse, Reynolds, Monk, Berman, & Kupfer, 1989), mood disorders (Steer & Beck, 1993), intelligence (Raven, 2003), as well as sport activities (self‐constructed), performance and motivation (Schuler & Prochaska, 2001). To ensure a regular sleep−wake rhythm (23:00 hours–07:00 hours), actigraphy (Cambridge Neurotechnologies) and a sleep diary (adapted from Saletu, Wessely, Grünerger, & Schultes, 1987) were applied 3 days prior to the baseline night and continued until the end of the testing period (day 6). Additionally, subjects were not allowed to ride a conventional bicycle throughout their study participation. As depicted in Figure 1, participants in both the AM‐PM‐AM and the PM‐AM‐PM group started with a baseline night (day 3, 23:00 hours–07:00 hours) to accustom them to sleeping with attached electrodes. On day 5, participants either participated in a 120 min inverse steering bicycle training session in the morning (AM‐PM‐AM) or in the evening (PM‐AM‐PM) followed by an initial straight‐line riding test session (TEST 1) and a subsequent wake/sleep retention interval. After 8 hr of wakefulness/sleep, subjects were re‐tested (TEST 2) in the evening/next morning and the following morning/evening (TEST 3) with another sleep/wake retention interval in between, i.e. every participant spent a wake as well as a sleep retention interval between the three test sessions with the AM‐PM‐AM group having slept after TEST 2 and the PM‐AM‐PM group having slept after TEST 1. The training session (5 × 20 min with 3 min rest in between blocks) aimed at learning to ride the inverse steering bicycle as good as possible. Instructions were standardized and designed to support a self‐paced exploratory learning by trial and error. Before every test session, subjective state and sleepiness were assessed by analogue scales (100 mm visual analogue scales [Analogue Scale for Evaluation of Sleepiness; ASES]; Folstein & Luria, 1973; Stanford Sleepiness Scale [SSS]; MacLean, Fekken, Saskin, & Knowles, 1992; “Mehrdimensionaler Befindlichkeitsfragebogen” [MDBF]; Steyer, Schwenkmezger, Notz, & Eid, 1997). In each test, subjects had to ride the inverse steering bicycle 5 × 30 m as straight as possible along a red line marked on the floor. A rotatory potentiometer mounted in the head tube of the inverse bike was used to assess riding accuracy by the mean standard deviation of the steering angle (SDSA [°] for rides 2–5; ride 1 served as warm‐up). In addition, speed, i.e. the mean riding time (s) for 4 × 30 m (rides 2–5; ride 1 served as warm‐up) straight‐line riding was measured by means of two video cameras.

### 2.3 Polysomnography

Polysomnography (PSG) was recorded using a Synamps amplifier (NeuroScan) during the 2 nights of sleep. PSG started at about 23:00 hours, and was terminated after 8 hr of time in bed (about 07:00 hours). Data were recorded referentially against a common reference at Cz and re‐referenced offline to contralateral mastoids (A1, A2). PSG recordings included 10 electroencephalogram channels (F3, Fz, F4, C3, C4, P3, Pz, P4, O1, O2), two horizontal electrooculogram (EOG) channels, two vertical EOG and two chin electromyogram channels were obtained at a sampling rate of 512 Hz. Sleep was automatically staged (Somnolyzer 24.9.7; Koninklijke Philips N.V.) and visually controlled by an expert scorer according to the American Academy of Sleep Medicine criteria (Iber, Ancoli‐Israel, Chesson, & Quan, 2007). Sleep spindles during N2 were detected automatically for electrode positions F3, F4, C3 and C4 (ASK analyser; The Siesta Group). Spindle detection was based on the following criteria: (a) 11–15 Hz band‐pass filtering; (b) amplitude > 25 µV; (c) duration > 0.5 s; and (d) controlling for muscle (30–40 Hz) and/or alpha (8–12 Hz) artefacts (Anderer et al., 2005; for more details please refer to Bothe et al., 2018). Within the spindle frequency band of 11–15 Hz, spindles were further divided into a slow (11–13 Hz) and a fast range (13–15 Hz). Because it has been suggested that spindles are involved in synaptic plasticity and long‐term potentiation processes (Rosanova & Ulrich, 2005), with spindle amplitudes being important for the extent of hippocampal‐neocortical memory reactivation (Bergmann, Mölle, Diedrichs, Born, & Siebner, 2012) and spindle duration being important for the optimal timing of information transfer into the cortex (Bonjean et al., 2011), SpA, i.e. mean spindle duration × mean spindle amplitude (Schabus et al., 2004), might be a sensible measure for reflecting state‐like or learning‐dependent consolidation processes (Lustenberger, Wehrle, Tüshaus, Achermann, & Huber, 2015). SpA was estimated by using an algorithm that gives an integer value for the envelope spanning the respective wave complexes within a 30‐s epoch, i.e. it captures the duration as well as the amplitude of identified spindles and thus reflects the power or intensity of the spindle process. For REM, total nighttime REM durations (min) were calculated for each participant and night. Furthermore, phasic (30‐s REM epoch with at least one certain rapid eye movement) and tonic (30‐s REM epoch without any certain rapid eye movement) REM episodes were detected automatically (REMalyzer; The Siesta Group) for all 10 electrode positions. Spectral analysis of consecutive 30‐s REM epochs (fast Fourier transform routine, Hanning window 10%, averages of 1‐s segments, max. frequency resolution 0.977 Hz) was performed for the theta frequency band (4–7 Hz; µV).

### 2.4 Statistical analyses

Statistical analyses were performed using IBM SPSS Statistics 24 (IBM). The significance level was set to *p* < .05. Effect sizes are provided as partial eta squared ($\eta_{p}^{2}$). Outliers were excluded from statistical analysis only when uni‐ or bivariate values did not meet the Grubb's criterion (Grubbs, 1950), the modified *z*‐score (Iglewicz & Hoaglin, 1993) and the Tukey fence of 2.2 × interquartile range (IQR) (Hoaglin & Iglewicz, 1987). The outlier analysis revealed extreme values for N2 SpA of one participant at electrode position C4 for the baseline as well as the intervening night of sleep. We did not detect any other extreme values for this participant at any of the remaining electrode positions (both nights). Thus, we excluded the participant only from calculations concerning N2 C4 SpA.

For investigating the effects of a night of intervening sleep and a wake retention interval on performance changes, performance values for TEST 1, 2 and 3 as well as performance change values (TEST 2 − TEST 1, TEST 3 − TEST 2 and TEST 3 − TEST 1) were calculated for SDSA and riding time. Subsequently, we conducted two‐factor analyses of variance (ANOVA) for repeated measures with the within‐subject factor TIME (TEST 1, TEST 2, TEST 3) and the between‐subject factor GROUP (AM‐PM‐AM versus PM‐AM‐PM) for SDSA and riding time. Post hoc independent and dependent samples *t*‐tests were applied when suitable. To control for possible differences in fatigue and mood between the two groups, independent‐sample *t*‐tests were applied. For investigating changes in sleep architecture from the baseline to the intervening night of sleep, two‐factor ANOVAs for repeated measures with the within‐subject factor NIGHT (baseline, intervening) and the between‐subject factor GROUP (AM‐PM‐AM, PM‐AM‐PM) were conducted separately for 10 macroarchitecture measures. For changes in sleep microarchitecture (N2 slow and fast SpA), three‐factor ANOVAs for repeated measures with the within‐subject factors NIGHT (baseline, intervening), LOCATION (F3, C3 for left hemisphere; F4, C4 for right hemisphere) and the between‐subject factor GROUP (AM‐PM‐AM, PM‐AM‐PM) were calculated. Post hoc dependent and independent sample *t*‐tests were applied. Pearson correlations (two‐tailed) were used to test whether overnight changes in gross motor performance linearly relate to N2 SpA, REM duration and spectral theta activity (tonic, phasic) during the baseline night and during the intervening night. Further, we tested whether these overnight changes in performance were related to the changes in sleep characteristics from the baseline night to the intervening night.

## 3 RESULTS

### 3.1 Behavioural data

Comparing the duration (min) subjects needed to reach the learning criterion of riding the inverse steering bicycle 3 × 30 m without dismounting, results for the training session revealed that both groups learned the task in a similar amount of time (*t*_23_ = 1.259, *p* = .221, $\eta_{p}^{2}$ = 0.064; AM‐PM‐AM: *M* = 89.58, *SD* = 21.46; PM‐AM‐PM: *M* = 75.92, *SD* = 31.41). Table 1 shows descriptive data for SDSA and riding time performance in both groups.

**Table 1:** Descriptive data for behavioural measures

	AM‐PM‐AM	PM‐AM‐PM	*t*	*p*	$\eta_{p}^{2}$
SDSA (°)					
TEST 1	11.60 ± 2.58	12.22 ± 4.39	−0.437	.666	0.008
TEST 2	15.54 ± 4.33	12.50 ± 4.32	1.793	.086	0.118
TEST 3	12.69 ± 3.67	10.98 ± 3.96	1.142	.265	0.052
Performance change (TEST2 − TEST1)	3.94 ± 3.09	0.28 ± 2.67	3.230	**.004**	0.303
Performance change (TEST3 − TEST2)	−2.85 ± 2.85	−1.52 ± 2.03	−1.372	.183	0.073
Performance change (TEST3 − TEST1)	1.09 ± 3.05	−1.24 ± 2.53	2.115	**.045**	0.157
Riding time (s)					
TEST 1	18.71 ± 5.97	15.75 ± 5.71	1.290	.209	0.065
TEST 2	25.74 ± 8.83	16.56 ± 5.82	3.130	**.005**	0.290
TEST 3	18.42 ± 5.81	13.69 ± 3.50	2.516	**.021**	0.243
Performance change (TEST2 − TEST1)	7.03 ± 6.29	0.80 ± 2.25	3.362	**.004**	0.320
Performance change (TEST3 − TEST2)	−7.32 ± 5.35	−2.87 ± 3.51	−2.509	**.019**	0.208
Performance change (TEST3 − TEST1)	−0.29 ± 6.02	−2.06 ± 3.93	0.892	.381	0.032

Note

Values are expressed as mean ± *SD*.

Abbreviation: SDSA, standard deviation of the steering angle.

Bold values denote statistical significance at the *p* < .05 level.

**Figure 2:**
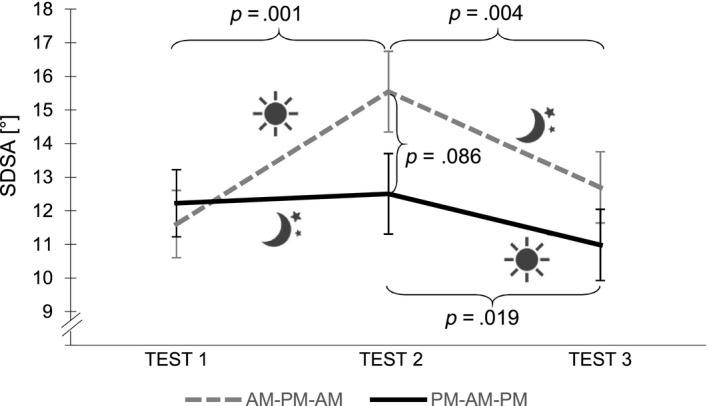
Standard deviation of the steering angle (SDSA) performance in AM‐PM‐AM and PM‐AM‐PM groups. Stabilization of SDSA performance and further improvement in the PM‐AM‐PM group. Deterioration with a subsequent performance increase in the AM‐PM‐AM group. High SDSA values indicate low performance. Error bars represent *SEM*

A significant main effect for TIME (*F*_2,48_ = 10.745, *p* < .001, $\eta_{p}^{2}$ = 0.309) and an interaction effect GROUP × TIME (*F*_2,48_ = 5.989, *p* = .005, $\eta_{p}^{2}$ = 0.200) were found for SDSA, indicating that the amount of change in riding accuracy throughout the three test sessions differed in the AM‐PM‐AM and PM‐AM‐PM groups (Figure 2). In the AM‐PM‐AM group, riding accuracy decreased significantly over the wake retention interval (*t*_12_ = −4.591, *p* = .001, $\eta_{p}^{2}$ = 0.637). Interestingly, riding accuracy levels went back to baseline scores after a night of intervening sleep (*t*_12_ = 3.606, *p* = .004, $\eta_{p}^{2}$ = 0.520). In the PM‐AM‐PM group, participants were able to stabilize riding accuracy after a night of intervening sleep (*t*_12_ = −0.379, *p* = .711, $\eta_{p}^{2}$ = 0.012) and to further improve performance across the subsequent wake retention interval (*t*_12_ = 2.702, *p* = .019, $\eta_{p}^{2}$ = 0.378). There were no significant group differences for TEST 1 (*t*_24_ = −0.437, *p* = .666, $\eta_{p}^{2}$ = 0.008) and TEST 3 (*t*_24_ = 1.142, *p* = .265, $\eta_{p}^{2}$ = 0.052). However, a trend for a group difference at TEST 2 (*t*_24_ = 1.793, *p* = .086, $\eta_{p}^{2}$ = 0.118) indicated that the PM‐AM‐PM group tended to perform more accurately than the AM‐PM‐AM group.

**Figure 3:**
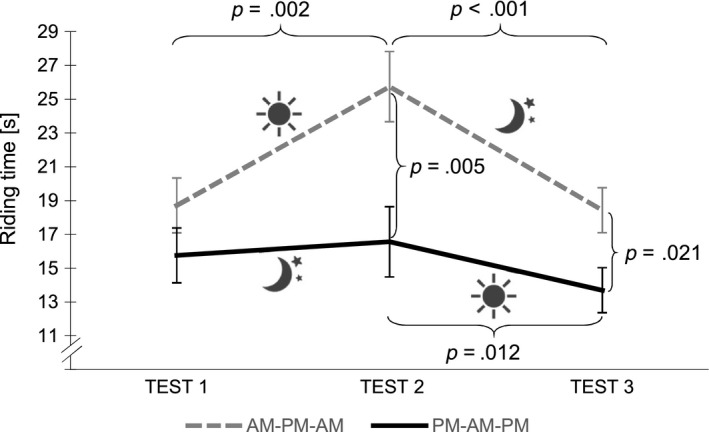
Riding time performance in AM‐PM‐AM and PM‐AM‐PM groups. Stabilization of riding time performance and further improvement in the PM‐AM‐PM group. Deterioration with a subsequent performance increase in the AM‐PM‐AM group. The PM‐AM‐PM group is significantly faster than the AM‐PM‐AM group after a night of sleep. This performance difference is preserved throughout TEST 3. High riding time values indicate low performance. Error bars represent *SEM*

A similar pattern was observed for riding time. In addition to the main effect for TIME (*F*_2,48_ = 16.169, *p* < .001, $\eta_{p}^{2}$ = 0.403) and an interaction effect GROUP × TIME (*F*_2,48_ = 5.851, *p* = .005, $\eta_{p}^{2}$ = 0.196), we also found a significant main effect for GROUP (*F*_1,24_ = 6.837, *p* = .015, $\eta_{p}^{2}$ = 0.222; Figure 3). Post hoc independent sample *t*‐tests showed that the PM‐AM‐PM group was significantly faster than the AM‐PM‐AM group at TEST 2 (*t*_24_ = 3.130, *p* = .005, $\eta_{p}^{2}$ = 0.290) and TEST 3 (*t*_24_ = 2.516, *p* = .021, $\eta_{p}^{2}$ = 0.243), i.e. subjects who spent a night of sleep right after training outperformed those subjects who had a wake retention interval after training, and continued to outperform them even after they had been given a full night of sleep. Similarly to SDSA performance, riding time in the AM‐PM‐AM group deteriorated (*t*_12_ = −4.031, *p* = .002, $\eta_{p}^{2}$ = 0.575) over the wake retention interval and was readjusted (*t*_12_ = 4.932, *p* < .001, $\eta_{p}^{2}$ = 0.700) to baseline performance levels over the following night of intervening sleep. In the PM‐AM‐PM group, a performance stabilization (*t*_12_ = −1.286, *p* = .223, $\eta_{p}^{2}$ = 0.121) occurred after a night of intervening sleep. This was followed by a significant performance improvement over the subsequent wake retention interval (*t*_12_ = 2.950, *p* = .012, $\eta_{p}^{2}$ = 0.420).

Taking possible differences in fatigue and mood (Multi‐dimensional Mood Questionnaire [MDMQ], ASES, SSS) into account, results revealed no group differences before TEST 1, 2 and 3 (Table 2).

**Table 2:** Fatigue and mood levels for AM‐PM‐AM and PM‐AM‐PM groups before TEST 1, 2 and 3

	TEST	AM‐PM‐AM	PM‐AM‐PM	*t*	*p*	$\eta_{p}^{2}$
MDMQ						
Affectivity (good – bad mood)	1	15.54 ± 3.57	17.54 ± 2.03	−1.756	.092	0.114
	2	16.62 ± 2.90	16.62 ± 3.45	0.000	1.000	< 0.001
	3	16.83 ± 2.29	18.23 ± 2.13	−1.582	.127	0.098
Sleepiness (awake – tired)	1	14.23 ± 2.80	13.85 ± 3.93	0.287	.777	0.003
	2	13.77 ± 4.09	15.08 ± 3.12	−0.917	.368	0.034
	3	14.75 ± 2.73	14.92 ± 3.38	−0.140	.890	0.001
Arousal (calm – nervous)	1	13.15 ± 3.21	15.62 ± 3.18	−1.965	.061	0.139
	2	15.08 ± 2.96	15.38 ± 4.25	−0.214	.832	0.002
	3	16.17 ± 2.95	16.62 ± 2.57	−0.407	.688	0.007
ASES						
Drive (active – passive)	1	27.38 ± 19.64	20.00 ± 10.64	1.192	.248	0.071
	2	34.92 ± 26.25	29.00 ± 19.42	0.654	.519	0.018
	3	31.33 ± 16.21	32.62 ± 21.96	−0.165	.870	0.001
Mood (sad – cheerful)	1	79.38 ± 14.23	80.46 ± 19.12	−0.163	.872	0.001
	2	77.08 ± 10.78	81.00 ± 14.24	−0.792	.436	0.025
	3	75.58 ± 12.30	82.69 ± 10.62	−1.551	.135	0.095
Sleepiness (awake – tired)	1	75.54 ± 18.53	83.46 ± 11.48	−1.310	.205	0.079
	2	68.31 ± 20.70	79.85 ± 18.32	−1.505	.145	0.086
	3	75.00 ± 14.18	75.00 ± 19.81	0.000	1.000	< 0.001
Participation (lethargic – compassionate)	1	32.62 ± 21.81	33.46 ± 21.60	−0.099	.922	< 0.001
	2	42.54 ± 28.46	30.77 ± 22.63	1.167	.255	0.054
	3	32.58 ± 22.08	35.85 ± 18.19	−0.405	.690	0.007
SSS						
Sleepiness (awake – tired)	1	2.00 ± 0.71	2.23 ± 1.24	−0.585	.564	0.014
	2	2.15 ± 1.52	1.92 ± 1.04	0.452	.655	0.008
	3	2.00 ± 0.74	2.23 ± 1.24	−0.561	.580	0.013

Note

Values are expressed as mean ± *SD*.

Abbreviations: ASES, Analogue Scale for Evaluation of Sleepiness; MDMQ, Multi‐dimensional Mood Questionnaire; SSS, Stanford Sleepiness Scale.

### 3.2 Sleep data

ANOVAs revealed significant main effects for total sleep time (*F*_1,24_ = 7.295, *p* = .012, $\eta_{p}^{2}$ = 0.233), sleep‐onset latency (*F*_1,24_ = 5.880, *p* = .023, $\eta_{p}^{2}$ = 0.197) and sleep efficiency (*F*_1,24_ = 6.004, *p* = .022, $\eta_{p}^{2}$ = 0.200), indicating differences in these measures between the baseline and the intervening night of sleep (Table 3).

**Table 3:** Sleep architecture

	AM‐PM‐AM						PM‐AM‐PM					
	*B*	*I*	Change	*t*	*p*	$\eta_{p}^{2}$	*B*	*I*	Change	*t*	*p*	$\eta_{p}^{2}$
TIB (min)	480.92 ± 5.40	481.50 ± 2.72	0.57 ± 7.51	−0.277	.786	0.006	480.04 ± 2.42	480.35 ± 5.15	0.31 ± 4.73	−0.234	.819	0.005
TST (min)	445.12 ± 37.11	461.92 ± 10.29	16.81 ± 28.95	−2.093	.058	0.267	435.46 ± 39.18	451.69 ± 17.29	16.23 ± 33.27	−1.759	.104	0.205
SOL (min)	15.65 ± 20.57	6.27 ± 4.51	−9.38 ± 19.72	1.716	.112	0.197	19.46 ± 17.82	12.88 ± 9.38	−6.58 ± 15.98	1.484	.164	0.155
EFF (%)	92.56 ± 7.76	95.94 ± 2.07	3.38 ± 6.29	−1.939	.076	0.239	90.70 ± 7.97	94.04 ± 3.54	3.34 ± 7.63	−1.577	.141	0.172
WASO (min)	19.50 ± 19.15	13.27 ± 9.60	3.38 ± 13.75	1.634	.128	0.182	24.42 ± 23.66	11.54 ± 6.01	−12.88 ± 22.97	2.022	.066	0.254
N1 (%)	11.85 ± 6.60	11.08 ± 4.63	−0.77 ± 5.93	0.471	.646	0.018	11.64 ± 4.29	10.74 ± 4.74	−0.90 ± 2.28	1.426	.179	0.145
N2 (%)	40.01 ± 10.33	37.04 ± 8.21	−2.97 ± 6.15	1.741	.107	0.202	40.22 ± 8.67	42.12 ± 7.90	1.91 ± 3.66	−1.877	.085	0.227
N3 (%)	31.71 ± 13.20	32.61 ± 9.73	0.90 ± 6.38	−0.508	.621	0.021	29.96 ± 12.47	28.91 ± 10.93	−1.06 ± 5.03	0.757	.464	0.046
REM (%)	16.34 ± 7.39	19.27 ± 5.86	2.93 ± 6.94	−1.523	.154	0.162	18.18 ± 4.76	18.23 ± 2.52	0.05 ± 3.29	−0.059	.954	<0.001
REM (min)	73.77 ± 34.60	88.96 ± 26.90	15.19 ± 32.00	−1.712	.113	0.196	79.69 ± 24.15	82.27 ± 11.23	2.58 ± 18.17	−0.511	.618	0.021

Note

Values are expressed as mean ± *SD*.

Abbreviations: B, baseline night; I, intervening night; Change, change in sleep parameters from baseline night to intervening night of sleep; TIB (min), time in bed; TST (min), total sleep time; SOL (min), sleep‐onset latency to N2; EFF (%), sleep efficiency; WASO (min), wake after sleep onset; N1 (%), percentage of sleep stage N1; N2 (%), percentage of sleep stage N2; N3 (%), percentage of sleep stage N3; REM (%), percentage of rapid eye movement sleep; REM (min), REM duration.

### 3.3 Sleep SpA

#### 3.3.1 General

**Figure 4:**
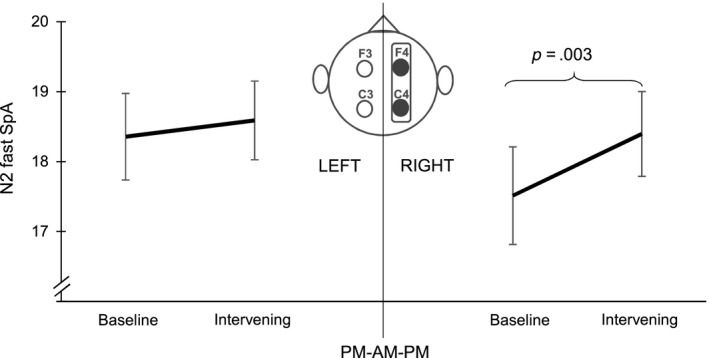
N2 fast spindle activity (SpA) change from the baseline night to the intervening night of sleep in the PM‐AM‐PM group. Participants showed a specific right hemispheric increase in N2 SpA (slow, fast and overall) from the baseline to the intervening night of sleep. Error bars represent *SEM*

ANOVA results for slow and fast SpA in the right hemisphere revealed significant main effects for NIGHT (SpA_slow_: *F*_1,24_ = 5.649, *p* = .026, $\eta_{p}^{2}$ = 0.191; SpA_fast_: *F*_1,23_ = 4.344, *p* = .048, $\eta_{p}^{2}$ = 0.159) as well as interaction effects NIGHT × GROUP (SpA_slow_: *F*_1,24_ = 5.466, *p* = .028, $\eta_{p}^{2}$ = 0.186; SpA_fast_: *F*_1,23_ = 6.564, *p* = .017, $\eta_{p}^{2}$ = 0.222). Furthermore, there was a significant main effect for LOCATION (SpA_fast_: *F*_1,23_ = 33.544, *p* < .001, $\eta_{p}^{2}$ = 0.593) as well as a significant interaction effect NIGHT × LOCATION × GROUP (SpA_fast_: *F*_1,23_ = 9.590, *p* = .005, $\eta_{p}^{2}$ = 0.294). Post hoc tests indicated that the PM‐AM‐PM group showed a specific increase in right hemispheric N2 SpA (slow and fast; Figure 4; Table 4) from the baseline night to the intervening night. Apart from a significant main effect for LOCATION (SpA_fast_: *F*_1,24_ = 43.253, *p* < .001, $\eta_{p}^{2}$ = 0.643) and a significant interaction effect NIGHT × LOCATION (SpA_fast_: *F*_1,24_ = 4.579, *p* = .043, $\eta_{p}^{2}$ = 0.160) indicating higher fast SpA at C3 compared with F3 in both the baseline and the intervening night of sleep (baseline_SpA fast_: *t*_25_ = −6.204, *p* < .001, $\eta_{p}^{2}$ = 0.606; intervening_SpA fast_: *t*_25_ = −6.358, *p* < .001, $\eta_{p}^{2}$ = 0.618), there were no significant results for the left hemisphere. Thus, further analyses of relations between N2 SpA and performance were limited to the right hemisphere.

**Table 4:** Spindle activity (overall, slow, fast) in the left and right hemisphere for both groups and nights

	AM‐PM‐AM						PM‐AM‐PM					
	*B*	*I*	Change	*t*	*p*	$\eta_{p}^{2}$	*B*	*I*	Change	*t*	*p*	$\eta_{p}^{2}$
Spindle activity (SpA)												
Left hemisphere	20.00 ± 3.88	19.84 ± 4.29	−0.16 ± 1.34	0.431	.674	0.015	20.58 ± 2.93	20.79 ± 2.62	0.21 ± 0.82	−0.919	.376	0.066
Slow	21.60 ± 3.70	20.96 ± 4.09	−0.63 ± 2.01	1.137	.278	0.097	21.29 ± 3.20	21.60 ± 2.67	0.31 ± 0.95	−1.172	.264	0.103
Fast	17.79 ± 2.66	17.58 ± 2.72	−0.20 ± 1.46	0.504	.623	0.021	18.36 ± 2.23	18.59 ± 2.03	0.23 ± 0.98	−0.857	.408	0.058
F3	19.96 ± 4.19	19.71 ± 4.56	0.26 ± 1.84	0.501	.625	0.021	19.98 ± 2.70	20.13 ± 2.46	−0.15 ± 0.95	−0.585	.569	0.028
Slow	21.45 ± 4.14	20.88 ± 4.47	0.57 ± 2.17	0.939	.366	0.068	20.75 ± 3.00	20.91 ± 2.61	−0.15 ± 1.07	−0.513	.617	0.021
Fast	16.97 ± 2.38	16.40 ± 2.57	0.57 ± 1.98	1.038	.320	0.082	17.24 ± 2.03	17.32 ± 1.97	−0.07 ± 1.21	−0.220	.829	0.004
C3	20.03 ± 3.75	19.97 ± 4.25	0.06 ± 1.15	0.188	.854	0.003	21.19 ± 3.46	21.45 ± 3.09	−0.26 ± 0.81	−1.166	.266	0.102
Slow	21.74 ± 3.51	21.04 ± 4.06	0.70 ± 1.42	1.038	.320	0.082	21.82 ± 3.88	22.29 ± 3.18	−0.46 ± 1.10	−1.519	.155	0.161
Fast	18.61 ± 3.15	18.77 ± 3.24	−0.16 ± 1.28	−0.466	.649	0.018	19.47 ± 2.64	19.86 ± 2.46	−0.39 ± 0.94	−1.504	.158	0.159
Right hemisphere	19.63 ± 3.66	19.62 ± 4.01	−0.01 ± 0.87	0.024	.981	< 0.001	20.32 ± 3.16	21.11 ± 2.93	0.81 ± 0.55	−5.074	**< .001**	0.701
Slow	20.74 ± 3.57	20.75 ± 3.81	0.01 ± 1.27	−0.020	.984	< 0.001	20.98 ± 3.56	21.92 ± 3.30	0.94 ± 0.67	−5.074	**< .001**	0.682
Fast	17.80 ± 2.32	17.71 ± 2.80	−0.09 ± 1.07	0.307	.764	0.008	18.00 ± 2.51	18.84 ± 2.19	0.88 ± 0.80	−3.810	**.003**	0.569
F4	19.72 ± 3.79	19.34 ± 3.93	0.38 ± 1.22	1.208	.250	0.108	19.78 ± 2.91	20.62 ± 2.50	−0.84 ± 0.78	−3.899	**.002**	0.559
Slow	20.88 ± 3.76	20.64 ± 3.68	0.24 ± 2.44	0.611	.553	0.030	20.49 ± 3.31	21.42 ± 2.80	−0.93 ± 0.94	−3.585	**.004**	0.517
Fast	17.11 ± 1.87	16.60 ± 2.21	0.51 ± 1.23	1.432	.178	0.146	16.74 ± 2.43	17.91 ± 1.97	−1.17 ± 1.07	−3.961	**.002**	0.567
C4	19.53 ± 3.67	19.90 ± 4.26	−0.38 ± 0.98	−1.378	.193	0.137	20.07 ± 2.58	20.81 ± 2.58	−0.73 ± 0.67	−3.781	**.003**	0.565
Slow	20.60 ± 3.81	20.86 ± 4.26	−0.26 ± 1.82	−0.507	.621	0.021	21.47 ± 4.29	22.42 ± 4.31	−0.95 ± 0.94	−3.651	**.003**	0.526
Fast	18.48 ± 2.94	18.81 ± 3.53	−0.33 ± 1.15	−1.029	.324	0.081	18.57 ± 1.81	19.10 ± 1.45	−0.54 ± 0.96	−1.938	.079	0.255

Note

Values are expressed as mean ± *SD*.

Abbreviations:* B*, baseline night; *I*, intervening night; Change, change in SpA from baseline night to intervening night of sleep; Slow, slow spindle activity (11–13 Hz); Fast, fast spindle activity (13–15 Hz).

Bold values denote statistical significance at the *p* < .05 level.

#### 3.3.2 PM‐AM‐PM group

**Figure 5:**
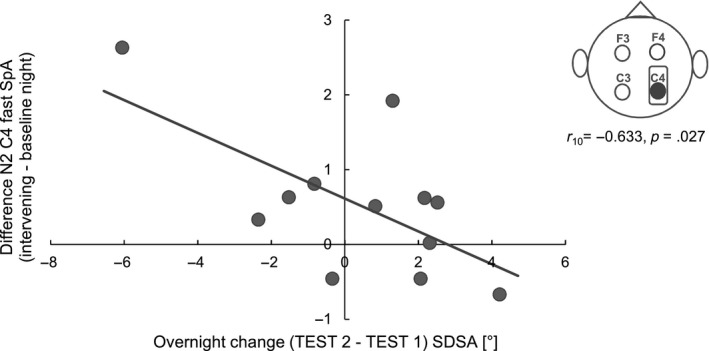
Riding accuracy (standard deviation of the steering angle; SDSA) and N2 fast spindle activity (SpA) changes from the baseline night to the intervening night of sleep. The higher the increase in C4 N2 fast SpA the less deterioration in performance after a night of intervening sleep

A higher increase in N2 fast SpA from the baseline night to the intervening night of sleep at C4 was associated with better stabilization of SDSA performance (*r*_10_ = −.633, *p* = .027; Figure 5). Riding accuracy (SDSA) was neither related to SpA during the baseline night nor to the intervening night of sleep, thus suggesting that the change in SpA was induced by learning to ride the inverse steering bicycle. Please note that one data point in Figure 5 might appear as a possible outlier. According to the outlier analysis described in “statistical analyses” this is not the case (Table S2 Supporting Information). However, if the data point is removed the correlation is no longer significant (*r*_11_ = −.267, *p* = .428). Furthermore, there were no significant correlations for slow SpA and riding accuracy.

**Figure 6:**
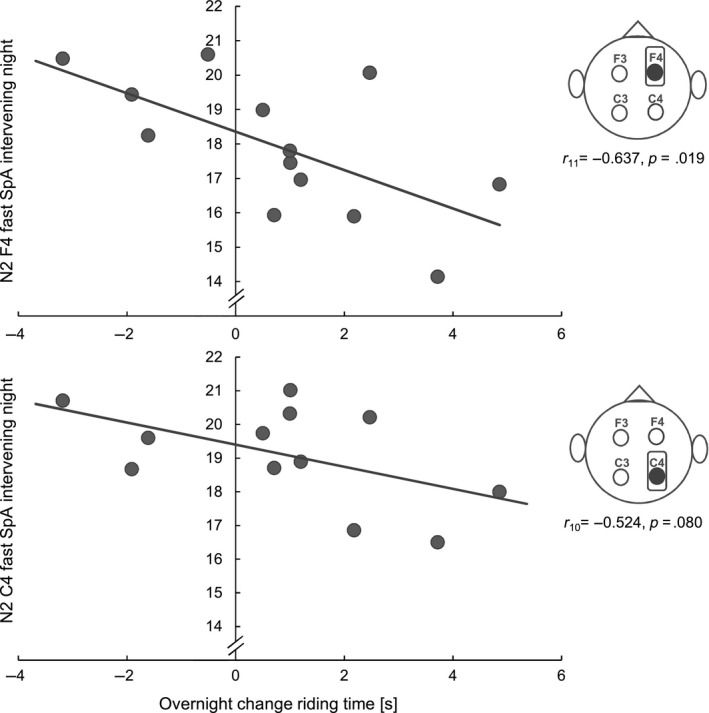
Riding time and N2 fast spindle activity (SpA). The higher the N2 fast SpA over the right hemisphere during the intervening night the less speed decline after sleep

For riding time, results showed that participants with higher N2 fast SpA over F4 during the intervening night had better stabilization of riding times over night (*r*_11_ = −.637, *p* = .019, Figure 6). There was also a trend for N2 fast SpA over C4 and riding time changes (*r*_10_ = −.524, *p* = .080), resulting in a significant correlation for right hemispheric N2 fast SpA with riding time changes over night (*r*_10_ = −.625, *p* = .030). However, a semi‐partial correlation (*r*_9_ = −.572, *p* = .066) revealed that the relation between intervening night and riding time changes was no longer significant when accounted for influences of SpA from the baseline night on the behavioural overnight change. This suggests that SpA during the learning night might reflect an underlying trait rather than training induced changes. Correlations with slow SpA and riding time did not reveal any significant results.

### 3.4 REM

In both groups, REM sleep durations did not significantly change between the baseline night and the intervening night of sleep (Table 3). Concerning REM sleep (duration, spectral phasic and tonic theta activity) and its relation to performance changes (SDSA, riding time), there were no significant correlations (Table S3 for REM duration and Table S4 in the supporting information for spectral theta activity).

## 4 DISCUSSION

This study investigated the effects of sleep on the consolidation of a gross motor adaptation task, i.e. riding and inverse steering bicycle. Results showed that a night of sleep (PM‐AM‐PM) right after training stabilized performance (accuracy and speed), while an equally long interval of wakefulness (AM‐PM‐AM) led to a significant deterioration in riding accuracy and speed. Furthermore, over the second retention interval, performance in the PM‐AM‐PM group improved significantly (8 hr wake), whereas performance in the AM‐PM‐AM group (8 hr sleep) was restored to post‐training performance levels, thereby eliminating the performance decline occurring following the initial wake interval. Regarding sleep, higher increases in fast SpA over C4 (from adaptation to intervening night) were related to better stabilization of accuracy, whereas more right hemispheric fast SpA (intervening night) was associated with a better stabilization of cycling speed. Our behavioural results are well in line with previous studies showing the importance of sleep for fine motor (Schönauer, Grätsch, & Gais, 2015; Stickgold, James, & Hobson, 2000) as well as gross motor learning (Brawn, Fenn, Nusbaum, & Margoliash, 2008; Malangré & Blischke, 2016). Moreover, the present results support the view that sleep may not enhance but rather stabilize motor adaptation skills (Pan & Rickard, 2015). According to recent findings in flies (aversive olfactory conditioning), memory stabilization might be promoted by the inhibition of dopaminergic activity during sleep (Berry, Cervantes‐Sandoval, Chakraborty, & Davis, 2015). Dopamine is required for the formation of long‐term potentiation, and is thought to facilitate motor learning and adaptation (Hosp & Luft, 2013; Nitsche et al., 2006). However, in states of prolonged wakefulness after learning, ongoing encoding of new motor information resulting in interference‐based forgetting may occur (Mednick, Cai, Shuman, Anagnostaras, & Wixted, 2011).

An interesting finding in the present study was the significant improvement in accuracy and speed across the wake retention interval of the PM‐AM‐PM group. Although some studies reported no further performance improvement across a wake interval following a night of sleep (Walker, Brakefield, Morgan, Hobson, & Stickgold, 2002), a similar (marginally significant) finding to ours has recently been reported by Malangré and Blischke (2016) using a gross motor sequence learning task. A possible interpretation would be an ongoing consolidation process. Because repetitive training, especially in a spaced design (i.e. long intervals between training sessions), is considered to help the formation of robust long‐term memory, the re‐tests after initial training can be considered as additional learning opportunities (Smolen, Zhang, & Byrne, 2016) causing re‐activation of the memory trace and triggering long‐term potentiation processes (Silva, 2003). It would have been interesting to see whether this sleep‐advantage would have remained over the long term as it has been shown for mirror tracing (Schönauer et al., 2015) and visuo‐motor skill training (Stickgold et al., 2000).

A further outcome of the present study was an increase in right hemispheric SpA from the adaptation to the intervening night in the PM‐AM‐PM group. Moreover, a higher increase in fast SpA over C4 (from adaptation to intervening night) was associated with better stabilization of steering accuracy. Additionally, higher fast SpA over the right hemisphere (intervening night) was associated with less decline in speed. This dominance of the right hemisphere in our results might be explained by the nature of the task. In the initial stages of learning how to ride an inverse steering bicycle, incoming information strongly and continuously contradicts the internal model of riding a normal bike. During training, the goal is to create an updated mental representation that integrates unexpected information, i.e. new environmental requirements, as accurately as possible. Recent research indicates that the key network for building and updating such an internal model is comprised of structures lying in the right hemisphere, including the right inferior parietal lobule (comparator of current evidence against model‐based expectations), the medial prefrontal cortex (error detection and conflict monitoring) and the anterior insula (integrating information to maintain an accurate mental model; Filipowicz et al., 2016). Sleep spindles have repeatedly been linked to procedural memory consolidation and are thought to reflect the local replay of memory traces acquired prior to sleep (Cox, Hofman, de Boer, & Talamini, 2014). Thus, the right hemispheric increase in SpA might reflect the reactivation of the network necessary for updating and expanding the mental model of “how to ride a bicycle” with “how to ride an inverse steering bicycle”. Interestingly, in another study where we used the inverse steering bicycle task in an adolescent sample, we reported a left hemispheric dominance (Bothe et al., 2018). The left hemisphere seems to be mainly involved in the control of complex movements, error processing and response inhibition. In the course of motor learning, activation in the right hemisphere decreases over time, whereas left hemispheric activation becomes more prominent with increased skill level (Serrien, Ivry, & Swinnen, 2006). It has to be noted that, in the adolescent sample, we made the task easier by providing supporting wheels. The difference in hemispheric dominance may therefore be explained by a faster learning process in the adolescent sample, possibly including less requirements in the model update domain and more requirements in the skill refinement domain. In a third study, we investigated gross motor adaptation learning in a nap paradigm (Hoedlmoser et al., 2015), and described that SpA and REM counteracted successful consolidation of the inverse steering bicycle task. In light of the recent results, it seems that a full night of sleep is necessary to at least stabilize performance in this task. Having a full night of sleep instead of a nap (more likely to be non‐habitual) is generally more likely to induce memory consolidation processes due to longer sleep durations (Schönauer et al., 2014), several consecutive non‐(N)REM and REM episodes, as well as less problems with falling and staying asleep at night time than during the day (King et al., 2017). Additionally, van Schalkwijk et al. (2017) examined sleep‐dependent memory consolidation (mirror tracing) by comparing a nap with a full night of sleep, and reported the latter to improve performance while the nap only stabilized motor adaptation skills. Another reason for the discrepancy with our current findings might be the timing of the sleep recording. While in the present study sleep was recorded directly after learning (initial training session and TEST 1), sleep recordings in the 2015 study took place 2 days after the initial training session and following an additional training phase, thus possibly reflecting different states in the learning process with different outcomes (e.g. cycles of memory reactivation, destabilization, degradation and reconsolidation; Stickgold & Walker, 2005).

Interestingly and contrary to our previous studies using the inverse steering bicycle (Bothe et al., 2018; Hoedlmoser et al., 2015), neither REM sleep duration nor theta activity during tonic and phasic REM episodes was related to overnight performance changes. According to the findings from Fogel, Ray, Binnie, and Owen (2015) on cognitive procedural skill acquisition (Tower of Hanoi task), REM might only be significantly involved on the night subjects become experts on the task, whereas sleep spindles are involved twice: (a) at an early stage when the strategy to perform the task is only starting to be acquired; and (b) after mastering the task for further refinement of the skill. Considering that our subjects were far from reaching expert level before the intervening night of sleep, the absence of REM effects might be plausible.

Despite furthering our knowledge about the role of sleep in gross motor adaptation learning, one potential limitation of the present study is that the baseline night was also used for acclimatization purposes. The occurrence of first night effects during acclimatization nights is a well‐known phenomenon (Curcio, Ferrara, Piergianni, Fratello, & Gennaro, 2004). Thus, using an actual baseline night without prior learning or a control night (as in Bothe et al., 2018) following a control learning task (e.g. riding a stationary bicycle) may have been more conclusive. Although participants showed significant differences in sleep‐onset latency, sleep efficiency and total sleep time between the baseline and the intervening night of sleep, it has to be noted that neither of the sleep stages, especially N2 and REM, seemed to be affected by these differences. Hence, sleep architecture seemed to be largely unchanged. Regarding sleep efficiency, we want to point out that, even in the baseline night, the mean percentage was about 92%, i.e. indicating a generally high quality of sleep during both nights (Beattie, Espie, Kyle, & Biello, 2015).

In summary, our results demonstrate that sleep facilitates the consolidation of a gross motor adaptation task, i.e. riding an inverse steering bicycle. A sleep interval right after gross motor adaptation training not only helped to stabilize but also led to further performance improvements over a subsequent wake retention interval. In contrast, staying awake after gross motor learning significantly deteriorated performance. However, participants were able to recover their post‐training performance levels after a night of sleep. Furthermore, right hemispheric fast N2 SpA was related to better stabilization of performance over night, thus possibly reflecting the ongoing process of updating the participants' mental model from “how to ride a bicycle” to “how to ride an *inverse* steering bicycle”.

## CONFLICT OF INTEREST

This was not an industry‐supported study. None of the authors has any financial conflict of interest.

## AUTHOR CONTRIBUTIONS

K.B. collected data, analysed the data, performed the statistical analysis, interpreted the results and drafted the manuscript. F.H., W.H.‐P. and E.J. collected and analysed the data. G.G. analysed the data. K.H. and J.B. designed the study, interpreted the results and drafted the manuscript. J.B. custom‐built the inverse steering bicycle. All authors read and approved the final manuscript.
