# Supplementary material for: Gross motor adaptation benefits from sleep after training
Source: J Sleep Res. 2019 Dec 23;29(5):e12961. doi: 10.1111/jsr.12961 (PMC7540033; doi:10.1111/jsr.12961)
Supplement: Supplementary file 1 [file JSR-29-e12961-s001.docx]

**Gross motor adaptation benefits from sleep after training**

Bothe, K.^1^, Hirschauer, F. ^1^, Wiesinger, H-.P. ^2^, Edfelder, J.M. ^2^, Gruber, G. ^3^, Hoedlmoser, K.^1*^, & Birklbauer, J. ^2*^

**^1^**Laboratory for Sleep, Cognition and Consciousness Research, Centre for Cognitive Neuroscience, University of Salzburg, Salzburg, Austria

^2^Department of Sport and Exercise Science, University of Salzburg, Salzburg, Austria

^3^Department of Psychiatry and Psychotherapy, Medical University of Vienna, Vienna, Austria.

***shared last authorship**

**Correspondence:**

Juergen Birklbauer, University of Salzburg,

Department of Sport and Exercise Science

Schlossallee 49,

5400 Hallein, Austria

Tel.: +43-662-8044-4885

e-mail: juergen.birklbauer@sbg.ac.at

Kerstin Hoedlmoser, University of Salzburg,

Centre for Cognitive Neuroscience, Laboratory

for Sleep, Cognition and Consciousness

Research, Hellbrunnerstrasse 34,

5020Salzburg, Austria.

Tel.: +43-662-8044-5143

e-mail: kerstin.hoedlmoser@sbg.ac.at

**Classification of learners and non-learners**

I) Factor analysis

By running an exploratory factor analysis with Varimax orthogonal rotation and an eigenvalue cut-off of 1.0, the five performance parameters – (i) ability to ride three runs of 30 m without dismounting, (ii) distance covered during the training session, as well as (iii) steering accuracy, (iv) riding time and (v) number of dismounts during TEST 1 – were reduced to a single factor, indicating the overall riding performance right after training. The Kaiser-Meyer-Olkin measure verified the sampling adequacy for the analysis, KMO = 0.727, with all KMO values for individual items being greater than 0.649. The extracted factor explained 71.1% of the variance, with the factor loadings depicted in Table S1. Those 26 subjects with the lowest factor scores were defined as learners, including all subjects being able to ride three runs of 30 m without dismounting and three further subjects who performed better than average at TEST 1. The cut-off between learners and non-learners was determined by the point of inflexion of the factor scores arranged in ascending order. This point of separation was further affirmed by a cluster analysis.

**Table S1**. Results of the exploratory factor analysis (N=35).

|  | Overall riding performance |
| --- | --- |
| Number of dismounts during TEST 1 | 0.920 |
| Riding time during TEST 1 | 0.900 |
| Ability to ride three runs of 30 m without dismounting | 0.864 |
| Distance covered during the training session | -0.795 |
| SDSA during TEST 1 | 0.722 |
| Eigenvalue | 3.55 |
| % variance | 71.09 |

II) Cluster analysis

To confirm the classification of learners and non-learners, a hierarchical agglomerative cluster analysis including all five performance variables was carried out with the unweighted pair-group method (average linkage between groups) as linkage measure and the squared Euclidean distance as similarity measure. Variables were standardized by z-score transformation. The distance coefficient markedly increased after stage 32, indicating three clusters. One cluster involved those 26 subjects that were previously identified as learners by factor analysis. The remaining two clusters contained the non-learners with one of them building an own cluster by showing the highest factor score (i.e. worst overall riding performance). The clustering process is illustrated by a dendrogram in Figure 1.

**Figure S1.** Dendrogram of the hierarchical cluster analysis (N=35).

**Table S2**. Outlier analysis for Figure 5 main article.

| OUTLIER ANALYSIS | | | | | |
| --- | --- | --- | --- | --- | --- |
|  |  | C4 N2 fast SpA baseline night | C4 N2 fast SpA intervening night | Difference (intervening night - baseline night) C4 N2 fast SpA | SDSA change |
| Grubb’s Criterion | Critical value z-score (2-sided) | 2.41 | 2.41 | 2.41 | 2.41 |
|  | Z-score participant | -1.27 | -0.15 | 2.18 | -2.31 |
| Modified  z-score | Critical value modified z-score (2-sided) | 4.33 | 4.33 | 4.33 | 4.33 |
|  | Modified z-score participant | -1.46 | -0.23 | 3.58 | -3.36 |
| Tukey Fence 2.2 x IQR | Upper fence | 27.32 | 27.30 | 4.13 | 11.44 |
|  | Lower fence | 10.03 | 10.17 | -3.52 | -7.19 |
|  | Value participant | 16.26 | 18.89 | 2.63 | -6.05 |

IQR: interquartile range

**Table S3**. Correlations between REM duration and performance change (SDSA, riding time) over the intervening night.

| CORRELATIONS REM x BEHAVIORAL MEASURES | | | | | | | | | | | | | | |
| --- | --- | --- | --- | --- | --- | --- | --- | --- | --- | --- | --- | --- | --- | --- |
|  |  | AM-PM-AM (n = 13) | | | | | |  | PM-AM-PM (n = 13) | | | | | |
|  |  | Change SDSA | | | Change riding time | | |  | Change SDSA | | | Change riding time | | |
|  |  | B | I | Change | B | I | Change |  | B | I | Change | B | I | Change |
|  |  |  |  |  |  |  |  |  |  |  |  |  |  |  |
| REM (min) | r | -0.260 | -0.154 | 0.166 | 0.364 | 0.203 | -0.219 |  | -0.028 | 0.255 | 0.195 | 0.245 | 0.296 | -0.142 |
|  | p | 0.039 | 0.615 | 0.589 | 0.221 | 0.507 | 0.473 |  | 0.927 | 0.401 | 0.524 | 0.421 | 0.326 | 0.644 |
| REM (%) | r | -0.274 | -0.149 | -0.224 | 0.355 | 0.189 | -0.219 |  | 0.055 | 0.267 | 0.125 | 0.310 | 0.338 | -0.190 |
|  | p | 0.366 | 0.627 | 0.463 | 0.234 | 0.537 | 0.473 |  | 0.857 | 0.377 | 0.748 | 0.303 | 0.259 | 0.535 |

B: baseline night; I: intervening night; Change: change in REM parameter from baseline night to intervening night of sleep

**Table S4.** Correlations between spectral theta activity (µV) during tonic/phasic REM sleep and overnight performance change in accuracy

(SDSA) and speed (riding time).

| SPECTRAL THETA ACTIVITY (µV) x BEHAVIORAL MEASURES | | | | | | | | | | | | | | | |
| --- | --- | --- | --- | --- | --- | --- | --- | --- | --- | --- | --- | --- | --- | --- | --- |
|  |  |  | AM-PM-AM (n = 13) | | | | | |  | PM-AM-PM (n = 13) | | | | | |
|  |  |  | Change SDSA | | | Change riding time | | |  | Change SDSA | | | Change riding time | | |
|  |  |  | B | I | Change | B | I | Change |  | B | I | Change | B | I | Change |
| F3 | phasic | r | 0.234 | 0.207 | -0.293 | -0.497 | -0.375 | 0.390 |  | 0.125 | 0.074 | 0.054 | 0.409 | 0.150 | -0.386 |
|  |  | p | 0.442 | 0.519 | 0.331 | 0.084 | 0.230 | 0.187 |  | 0.685 | 0.811 | 0.861 | 0.165 | 0.624 | 0.192 |
|  | tonic | r | 0.172 | 0.217 | -0.228 | -0.426 | -0.526 | 0.413 |  | 0.165 | 0.099 | 0.031 | 0.294 | 0.102 | -0.236 |
|  |  | p | 0.573 | 0.477 | 0.454 | 0.147 | 0.065 | 0.161 |  | 0.589 | 0.748 | 0.920 | 0.330 | 0.741 | 0.437 |
| Fz | phasic | r | 0.227 | 0.164 | -0.291 | -0.440 | -0.358 | 0.351 |  | 0.168 | 0.028 | -0.029 | 0.575 | 0.063 | -0.565 |
|  |  | p | 0.455 | 0.610 | 0.336 | 0.132 | 0.253 | 0.239 |  | 0.622 | 0.928 | 0.933 | 0.064 | 0.839 | 0.070 |
|  | tonic | r | 0.199 | 0.199 | -0.254 | -0.368 | -0.481 | 0.374 |  | 0.228 | 0.067 | 0.078 | 0.394 | 0.063 | -0.261 |
|  |  | p | 0.516 | 0.515 | 0.402 | 0.216 | 0.096 | 0.208 |  | 0.500 | 0.829 | 0.810 | 0.230 | 0.837 | 0.413 |
| F4 | phasic | r | 0.284 | 0.170 | -0.321 | -0.449 | -0.337 | 0.334 |  | 0.152 | 0.118 | 0.068 | 0.404 | 0.083 | -0.384 |
|  |  | p | 0.348 | 0.597 | 0.285 | 0.124 | 0.284 | 0.265 |  | 0.621 | 0.702 | 0.826 | 0.171 | 0.786 | 0.195 |
|  | tonic | r | 0.211 | 0.191 | -0.246 | -0.380 | -0.477 | 0.363 |  | 0.220 | 0.152 | 0.029 | 0.247 | 0.071 | -0.211 |
|  |  | p | 0.490 | 0.532 | 0.418 | 0.200 | 0.099 | 0.223 |  | 0.471 | 0.619 | 0.924 | 0.415 | 0.819 | 0.488 |
| C3 | phasic | r | 0.124 | -0.079 | -0.182 | -0.315 | -0.435 | 0.288 |  | 0.196 | 0.223 | 0.004 | 0.436 | 0.335 | -0.325 |
|  |  | p | 0.687 | 0.797 | 0.552 | 0.295 | 0.138 | 0.340 |  | 0.522 | 0.465 | 0.989 | 0.136 | 0.264 | 0.278 |
|  | tonic | r | 0.113 | -0.009 | -0.215 | -0.260 | -0.405 | 0.357 |  | 0.286 | 0.272 | -0.008 | 0.200 | 0.323 | -0.181 |
|  |  | p | 0.714 | 0.977 | 0.480 | 0.390 | 0.169 | 0.232 |  | 0.344 | 0.369 | 0.979 | 0.513 | 0.282 | 0.553 |
| Cz | phasic | r | 0.208 | 0.087 | -0.259 | -0.354 | -0.376 | 0.270 |  | 0.189 | 0.241 | 0.020 | 0.468 | 0.362 | -0.338 |
|  |  | p | 0.495 | 0.779 | 0.392 | 0.236 | 0.206 | 0.372 |  | 0.536 | 0.428 | 0.948 | 0.107 | 0.224 | 0.259 |
|  | tonic | r | 0.184 | 0.151 | -0.248 | -0.268 | -0.307 | 0.320 |  | 0.268 | 0.274 | 0.003 | 0.276 | 0.333 | -0.205 |
|  |  | p | 0.548 | 0.622 | 0.414 | 0.376 | 0.307 | 0.287 |  | 0.376 | 0.365 | 0.992 | 0.361 | 0.266 | 0.503 |
| C4 | phasic | r | 0.168 | -0.177 | -0.213 | -0.365 | -0.093 | 0.268 |  | 0.029 | 0.132 | 0.114 | 0.366 | 0.387 | -0.273 |
|  |  | p | 0.583 | 0.562 | 0.485 | 0.220 | 0.763 | 0.376 |  | 0.924 | 0.668 | 0.710 | 0.219 | 0.191 | 0.367 |
|  | tonic | r | 0.165 | -0.116 | -0.231 | -0.263 | -0.044 | 0.307 |  | 0.140 | 0.202 | 0.057 | 0.176 | 0.371 | -0.161 |
|  |  | p | 0.591 | 0.706 | 0.448 | 0.386 | 0.887 | 0.308 |  | 0.648 | 0.508 | 0.853 | 0.565 | 0.212 | 0.600 |
| P3 | phasic | r | -0.021 | -0.268 | -0.057 | -0.485 | -0.361 | 0.332 |  | 0.021 | 0.140 | 0.022 | 0.448 | 0.303 | -0.377 |
|  |  | p | 0.946 | 0.375 | 0.852 | 0.093 | 0.225 | 0.268 |  | 0.950 | 0.649 | 0.949 | 0.167 | 0.314 | 0.253 |
|  | tonic | r | -0.019 | -0.235 | -0.134 | -0.386 | -0.320 | 0.381 |  | 0.058 | 0.198 | 0.126 | 0.234 | 0.284 | -0.163 |
|  |  | p | 0.951 | 0.440 | 0.663 | 0.193 | 0.287 | 0.198 |  | 0.865 | 0.518 | 0.697 | 0.489 | 0.346 | 0.613 |
| Pz | phasic | r | -0.001 | -0.277 | -0.081 | -0.439 | -0.296 | 0.291 |  | 0.084 | 0.198 | -0.026 | 0.473 | 0.346 | -0.399 |
|  |  | p | 0.997 | 0.359 | 0.792 | 0.133 | 0.326 | 0.335 |  | 0.807 | 0.516 | 0.940 | 0.142 | 0.247 | 0.224 |
|  | tonic | r | 0.013 | -0.189 | -0.161 | -0.317 | -0.271 | 0.344 |  | 0.129 | 0.264 | 0.098 | 0.280 | 0.325 | -0.183 |
|  |  | p | 0.965 | 0.537 | 0.600 | 0.290 | 0.370 | 0.250 |  | 0.705 | 0.383 | 0.761 | 0.404 | 0.278 | 0.568 |
| P4 | phasic | r | -0.027 | -0.273 | -0.047 | -0.341 | -0.343 | 0.258 |  | -0.076 | 0.180 | 0.089 | 0.368 | 0.302 | -0.320 |
|  |  | p | 0.931 | 0.367 | 0.880 | 0.254 | 0.251 | 0.394 |  | 0.825 | 0.555 | 0.795 | 0.266 | 0.316 | 0.338 |
|  | tonic | r | 0.007 | -0.222 | -0.158 | -0.244 | -0.251 | 0.327 |  | -0.042 | 0.233 | 0.170 | 0.167 | 0.265 | -0.132 |
|  |  | p | 0.981 | 0.466 | 0.606 | 0.422 | 0.409 | 0.276 |  | 0.903 | 0.443 | 0.597 | 0.623 | 0.382 | 0.683 |
| O1 | phasic | r | 0.074 | -0.090 | -0.108 | -0.134 | -0.294 | 0.107 |  | -0.255 | 0.129 | 0.254 | 0.010 | 0.205 | -0.025 |
|  |  | p | 0.809 | 0.770 | 0.725 | 0.663 | 0.330 | 0.728 |  | 0.400 | 0.675 | 0.403 | 0.973 | 0.502 | 0.935 |
|  | tonic | r | 0.086 | -0.072 | -0.229 | -0.086 | -0.250 | 0.229 |  | -0.233 | 0.126 | 0.213 | -0.052 | 0.195 | -0.007 |
|  |  | p | 0.780 | 0.815 | 0.451 | 0.780 | 0.409 | 0.452 |  | 0.443 | 0.681 | 0.485 | 0.866 | 0.523 | 0.981 |
| O2 | phasic | r | 0.040 | -0.111 | -0.100 | -0.186 | -0.223 | 0.082 |  | -0.275 | 0.053 | 0.280 | 0.013 | 0.233 | -0.035 |
|  |  | p | 0.897 | 0.718 | 0.745 | 0.543 | 0.464 | 0.790 |  | 0.364 | 0.864 | 0.355 | 0.967 | 0.444 | 0.909 |
|  | tonic | r | 0.109 | -0.115 | -0.224 | -0.041 | -0.132 | 0.166 |  | -0.284 | 0.067 | 0.246 | -0.095 | 0.236 | 0.009 |
|  |  | p | 0.724 | 0.707 | 0.462 | 0.893 | 0.666 | 0.587 |  | 0.347 | 0.828 | 0.419 | 0.758 | 0.438 | 0.977 |

B: baseline night; I: intervening night; Change: change in REM parameter from baseline night to intervening night of sleep; phasic: spectral theta

activity (µV) during phasic REM; tonic: spectral theta activity (µV) during tonic REM; change: change in spectral theta activity (µV) from the

baseline to the intervening night of sleep
